# Evidence for a consistent use of external cues by marine fish larvae for orientation

**DOI:** 10.1038/s42003-022-04137-7

**Published:** 2022-12-02

**Authors:** Igal Berenshtein, Robin Faillettaz, Jean-Oliver Irisson, Moshe Kiflawi, Ulrike E. Siebeck, Jeffery M. Leis, Claire B. Paris

**Affiliations:** 1 Rosenstiel School of Marine and Atmospheric Science University of Miami 4600 Rickenbacker Causeway, Miami, FL 33149 USA; 2 Cooperative Institute for Marine and Atmospheric Studies, Rosenstiel School of Marine and Atmospheric Science, University of Miami, 4600 Rickenbacker Causeway, Miami, FL 33149 USA; 3 Department of Marine Biology, Leon H. Charney School of Marine Sciences, University of Haifa, Mt. Carmel, 3498838 Haifa, Israel; 4 Centre National de la Recherche Scientifique, Laboratoire d’Océanographie de Villefranche-sur-Mer (LOV), Sorbonne Universités, UPMC University Paris 06, Villefranche-sur-Mer, France; 5 EDECOD (Ecosystem Dynamics and Sustainability), IFREMER, INRAE, Institut Agro, Lorient, France; 6 Laboratoire d’Océanographie de Villefranche, LOV, Sorbonne Université, CNRS, F-06230 Villefranche-sur-Mer, France; 7 Department of Life-Sciences, Ben-Gurion University of the Negev, POB 653, 84105 Beer-Sheva, Israel; 8 The Interuniversity Institute for Marine Sciences of Eilat, Eilat, 88103 Israel; 9 Laboratory for Visual Neuroethology, School of Biomedical Sciences, University of Queensland, St Lucia, QLD 4072 Australia; 10 Ecology and Biodiversity Centre, Institute for Marine and Antarctic Studies, University of Tasmania, Hobart, TS 7007 Australia; 11 Ichthyology, Australian Museum Research Institute, Sydney, NSW 2001 Australia

**Keywords:** Behavioural ecology, Animal migration

## Abstract

The larval stage is the main dispersive process of most marine teleost species. The degree to which larval behavior controls dispersal has been a subject of debate. Here, we apply a cross-species meta-analysis, focusing on the fundamental question of whether larval fish use external cues for directional movement (i.e., directed movement). Under the assumption that directed movement results in straighter paths (i.e., higher mean vector lengths) compared to undirected, we compare observed patterns to those expected under undirected pattern of Correlated Random Walk (CRW). We find that the bulk of larvae exhibit higher mean vector lengths than those expected under CRW, suggesting the use of external cues for directional movement. We discuss special cases which diverge from our assumptions. Our results highlight the potential contribution of orientation to larval dispersal outcomes. This finding can improve the accuracy of larval dispersal models, and promote a sustainable management of marine resources.

## Abstract (web summary)

In the majority of tested marine teleost fish species, larvae rely on external cues for orientation. This finding is important to understand larval behaviour and its implementation in biophysical dispersal models used for sustainable management and conservation of marine resources.

## Introduction

The larval phase is the main dispersive stage of most demersal teleost marine fishes, controlling population dynamics and shaping connectivity patterns. As such, it plays a key role in large-scale ecological processes such as gene flow and biogeography^1^. The dispersal of larval fish is governed by two main mechanisms, ocean currents and larval behavior^1^. Although our understanding of ocean currents has greatly improved in the past decades allowing better reconstruction and prediction, a proper understanding of larval behavior has been more challenging due to a high degree of uncertainty and inter- and intra-specific variability in larval traits^2^.

In the past two decades, multiple studies demonstrated substantial swimming and orientation capabilities for larval fish of various species, which can affect their dispersal outcome^3,4^. However, most of these empirical studies focused on a single species at a single location, such that a generalized cross-species inference has never been attempted. This might be the reason why larval behavior is not implemented in most biophysical larval dispersal models^5^. Here, we apply a cross-species meta-analysis focusing on the fundamental behavioral trait of larval directional swimming.

Recent studies have repeatedly demonstrated that fish larvae influence their dispersal by swimming directionally^3^. Directional movement is a central component in animal movement ecology^6,7^, referring to the tendency of an individual to move along a straight path^3,7^. A movement is considered directional if it has a significant directional precision (or mean vector length), based on Rayleigh’s test of uniformity^8^. Directional movement is different from directed or oriented movement, in which by definition, there is an inherent use of external cues^9^. Similarly, unoriented movement is a situation in which there is no use of external cues for orientation.

Directional movement can be achieved using internal stimuli or with reference to external cues, with only the latter representing oriented movement^6^. It is currently unclear which type is used by fish larvae. This is critical since larval dispersal is a key process governing demographic connectivity, gene flow and biogeography of marine populations^10^. Incorrect representation of larval orientation in biophysical models can lead to inaccurate estimations of larval transport and connectivity^11^. To test whether fish larvae use external cues for directional movement, we compare observed movement patterns to those expected under a strict use of internal cues. Our analyses are based on the simplifying assumptions that a strict use of internal cues is expressed in a Correlated Random Walk (CRW) process, whereby consecutive movement directions or ‘bearings’ are auto-correlated^11^, and that oriented movement patterns, measured across multiple time steps (>20), are more directional (i.e., straighter paths characterized by higher mean vector lengths) compared to unoriented patterns^12,13^. Under these assumptions, we find a robust support for the use of external cues by fish larvae, both at the individual and at the species levels. We discuss cases that diverge from these assumptions, for which our methodology is inappropriate.

Directional movement has been demonstrated in fish larvae of more than 20 species^3,14–20^. The studied larvae were mostly wild larvae captured by light traps, although some were reared (Table 1). All were in the post-flexion stage of development, meaning the caudal fin was formed, and they had the ability to swim at speeds that meant they were moving in an inertial hydrodynamic environment^21,22^. Two formats of field trials were used to assess larval directional movement: (i) tracking larvae by scuba-divers (*Scuba-Following*^23^) and (ii) video recording of larvae inside the Drifting In Situ Chamber (*DISC*^24^). In both types of trials, the observed precision could be achieved by utilizing external (i.e., oriented) or internal (i.e., unoriented) cues. The difference relies on the orientation mechanism: larvae that utilize internal cues may use their proprioceptive system, similar to a gyroscope^25^, or simply keep moving toward the same general direction due to inertia^26^, giving rise to CRW^11^. In contrast, truly-orienting larvae use external cues such as the earth’s magnetic field^27^, resulting in patterns such as Biased Random Walk (BRW), whereby bearings correlate with a fixed external direction^6,11^. The distinction between the two is critical since external cues enable a more persistent directional movement over time, and it allows for corrections should the larvae be displaced^6^.

**Table 1:** Species used for the meta-analysis and relevant statistics comparing ${\hat{R}}_{\theta }$ quantile distribution with the null ${R}_{{\theta }_{0}^{{vm}}}$ and ${R}_{{\theta }_{0}^{r}}$quantile distributions using chi-square (*X*^2^*)* goodness-of-fit test.

Species	Abbreviation	Family	Mean *△R*_*θ*_	95% CI	*X*^2^_*CRW-vm*_	*Cohen’s W*_*CRW-vm*_	*X*^2^_*CRW-r*_	*Cohen’s W*_*CRW-r*_	Number of individuals	Method	*N*_*obs*_ per individual	Citation	Location
*Trachurus spp*.	Tra_ spp.	Carangidae	0.59	0.1	–	–	–	–	2	DISC	180	^2^	MED
*Chromis atripectoralis_group*	Chr_atri_group	Pomacentridae	0.43	0.1	2.6	0.84	16^**^	2.6	23	DISC	90	^5^	GBR
*Chromis chromis*	Chr_chro	Pomacentridae	0.42	0.05	150^**^	1.52	165^**^	1.59	65 (67)	DISC	180	^2^	MED
*Chromis atripectoralis_ ind*	Chr_atri_ind	Pomacentridae	0.4	0.05	10^**^	0.34	65^**^	0.9	83 (86)	DISC	90	^5^	GBR
*Oblada melanura*	Obl_mela	Sparidae	0.35	0.08	66^**^	1.73	66^**^	1.73	22	DISC	180	^2^	MED
*Premnas biaculeatus*	Pre_biac	Pomacentridae	0.32	0.1	19.8^**^	1	24.5^**^	1	23 (24)	DISC	300	^1^	GOA
*Diplodus annularis*	Dip_annu	Sparidae	0.32	0.07	90^**^	1.73	90^**^	1.73	30 (35)	DISC	180	^2^	MED
*Spicara smaris*	Spi_smar	Sparidae	0.3	0.062	86.9^**^	1.6	94.2^**^	1.67	34	DISC	180	^2^	MED
*Boops boops*	Boo_boop	Sparidae	0.28	0.1	–	–	–	–	7	DISC	180	^2^	MED
*Spondyliosoma cantharus*	Spo_cant	Sparidae	0.226	0.176	–	–	–	–	6	DISC	180	^2^	MED
*Pomacentrus lepidogenys*	Pom_lepi	Pomacentridae	0.174	0.037	20.3^**^	0.6	56.6^**^	1	54 (59)	Sc-Fl	21	^6^	GBR
*Chaetodon plebeius*	Cha_pleb	Chaetodontidae	0.167	0.058	15.6^**^	0.7	25.9^**^	0.96	28	Sc-Fl	21	^6^	GBR
*Epinephelus fuscoguttatus*	Epi_fusc	Serranidae	0.136	0.121	–	–	–	–	9 (12)	Sc-Fl	21	^7^	SCS
*Eleutheronema tetradactylum*	Ele_tetr	Polynemidae	0.132	0.056	6.1^*^	0.54	15.3^**^	0.85	21 (27)	Sc-Fl	21	^8^	SCS
*Chrysiptera rollandi*	Chr_roll	Pomacentridae	0.11	0.038	12.3^**^	0.49	24.3^**^	0.69	51 (56)	Sc-Fl	21	^6^	GBR
*Caesio cuning*	Cae_cuni	Caesionidae	0.103	0.058	–	–	–	–	10	Sc-Fl	21	^6^	GBR
*Chromis atripectoralis_group*	Chr_atri_group	Pomacentridae	0.089	0.028	–	–	–	–	15	Sc-Fl	21	^5^	GBR
*Neopomacentrus cyanomos*	Neo_cyan	Pomacentridae	0.085	0.036	16.7^**^	0.44	15.6^**^	0.43	85 (98)	Sc-Fl	21	^6^	GBR
*Platax teira*	Pla_teir	Ephippidae	0.064	0.174	–	–	–	–	7	Sc-Fl	21	^7^	SCS
*Caranx ignobilis*	Car_igno	Carangidae	0.063	0.082	-–	–	–	–	12 (17)	Sc-Fl	21	^9^	SCS
*Epinephelus coioides*	Epi_coio	Serranidae	0.061	0.076	–	–	–	–	15 (22)	Sc-Fl	21	^7^	SCS
*Chromis atripectoralis_ ind*	Chr_atri_ind	Pomacentridae	0.047	0.030	1.8	0.18	8.14^*^	0.38	56 (62)	Sc-Fl	21	^6^	GBR
*Amblyglyphidodon curacao*	Amb_cura	Pomacentridae	0.043	0.045	1.6	0.2	25.6^**^	0.7	55 (65)	Sc-Fl	21	^10^	GBR
*Chaetodon aureofasciatus*	Cha_aure	Chaetodontidae	0.030	0.048	0.9	0.1	24.4^**^	0.8	42 (48)	Sc-Fl	21	^6^	GBR
*Diplodus annularis*^*s*^	Dip_annu	Sparidae	0.32	0.07	–	–	–	–	4	DISC	21	^47^	MED
*Spicara_smaris* ^*s*^	Spi_smar	Sparidae	0.256	0.06	–	–	–	–	10	DISC	21	^2^	MED
*Boops boops* ^*s*^	Boo_boop	Sparidae	0.25	0.13	–	–	–	–	3	DISC	21	^2^	MED
*Premnas biaculeatus* ^*s*^	Pre_biac	Pomacentridae	0.24	0.072	17.1^**^	0.92	17.1^**^	0.92	20	DISC	21	^4^	GOA
*Chromis atripectoralis_ ind* ^*s*^	Chr_atri_ind	Pomacentridae	0.23	0.042	25.3^**^	0.59	40.4^**^	0.74	74	DISC	21	^5^	GBR
*Chromis chromis*^*s*^	Chr_chro	Pomacentridae	0.21	0.048	–	–	–	–	11	DISC	21	^47^	MED
*Spondyliosoma cantharus*^*s*^	Spo_cant	Sparidae	0.15	0.154	–	–	–	–	4	DISC	21	^47^	MED
*Chromis atripectoralis_group*^*s*^	Chr_atri_group	Pomacentridae	0.13	0.062	0.4	0.14	5.1	0.5	20	DISC	21	^5^	GBR

*The data in^5^ are from trials with individuals and groups of *Chromis atripectoralis* using the *DISC*.

^*S*^Subsamples of DISC trials to *N*_*obs*_ = 21 for comparison with Scuba-Following trials.

The following statistics are provided: *ΔR*: the difference between the observed mean vector (${\hat{R}}_{\theta }$*)* and that expected under *CRW* (${R}_{{\theta }_{0}^{{vm}}}$) (*ΔR* = ${\hat{R}}_{\theta }$*-* ${\bar{R}}_{{\theta }_{0}^{{vm}}}$), *X*^*2*^ test statistics value, Cohen’s W effect size value. One or two asterisks on the *X*^*2*^ value represent significant *X*^*2*^ tests with *P* < 0.05 or *P* < 0.01, respectively. Method abbreviations: Drifting In-Situ Chamber (*DISC*) and *Scuba-Following* (*Sc-Fl*); locations: GBR- Lizard Island, Great Barrier Reef, Australia; MED- Mediterranean Sea, France; SCS- South China Sea, Taiwan Island; GOA – Gulf of Aqaba, Red Sea. The number of directional (Rayleigh’s test, *p* < 0.05) individuals per species is given as well as the total number of individuals (in brackets). Numbers in brackets are absent in species for which all individuals were directional. Rows are sorted according to descending order of *ΔR* values. Subsampled *DISC* trials (*N*_*obs*_ = 21) are given in the bottom part of the table, separated from the rest of the groups with a horizonal line, and notated with (^s^).

In the present work, we show that the mean vector lengths larval-fish field trials were significantly higher that those expected under CRW both at the individual and at the species levels, providing supporting evidence the use of external cues for directional movement by fish larvae.

## Results

**Fig. 1:**
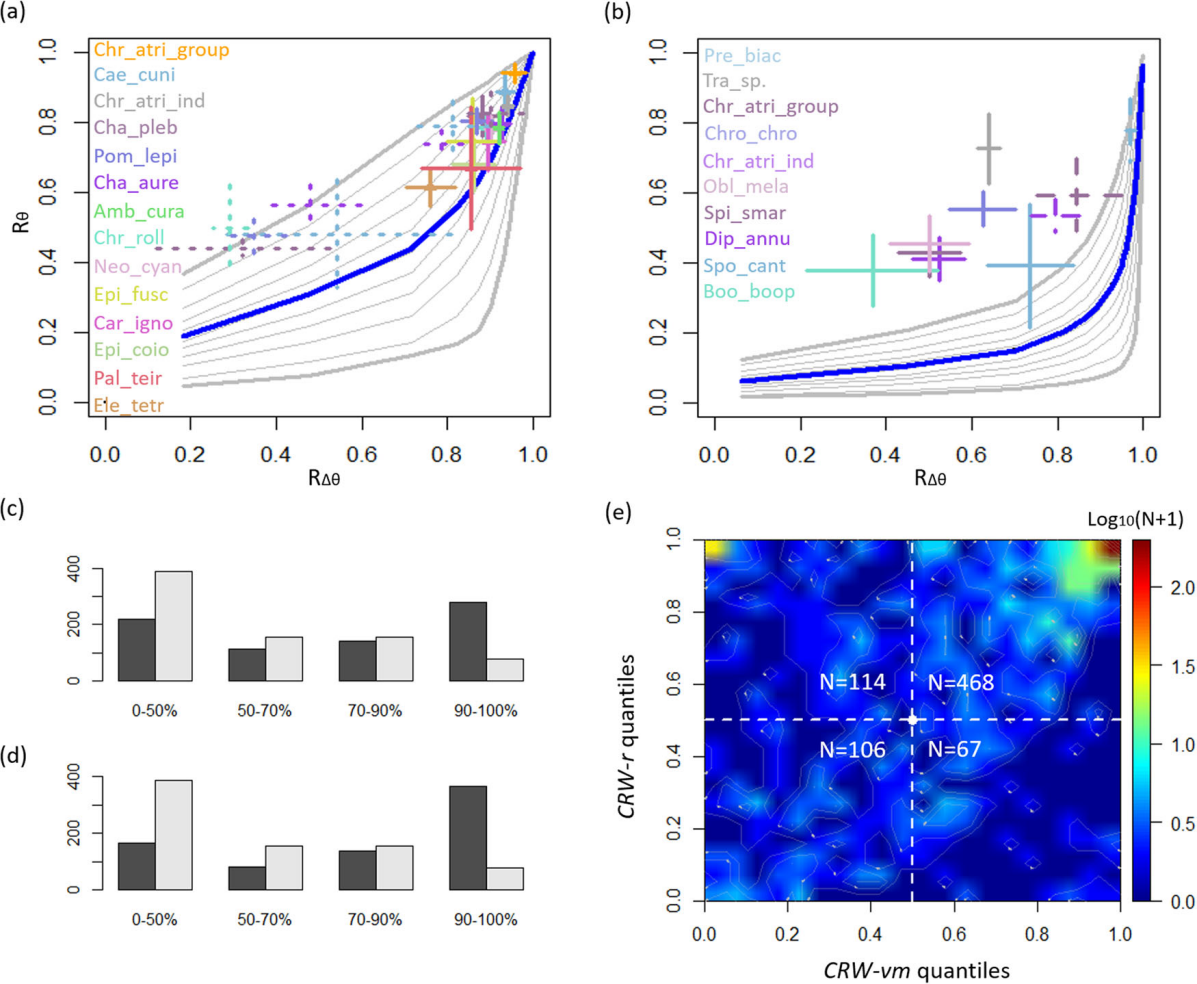
Results of Correlated Random Walk-von Mises (*CRW-vm*) and Correlated Random Walk resampling (*CRW-r*). *CRW-vm* analysis at the species level based on the diagram in Fig. 2c for the *scuba-following* trials (**a**), and for the *DISC* trials (**b**) with various number of samples-per-trial: *N*_*obs*_ = 21 (a), 300, 90, and 180 (**b**). Crosses represent means ± 95% CI of the observed (*R*_*Δθ*_, *R*_*θ*_) pooled by species. Crosses with dashed lines in **b** represent species with *N*_*obs*_ different than 180, see Table 1 for more details. The 5th, 10th, 20th,…., 90th, and 95th quantiles of the ${R}_{{\theta }_{0}^{{vm}}}$ distribution is represented in grey contours, with thicker contours for the 5th and 95th quantiles. Thick blue line in **a** and **b** represents ${\bar{R}}_{{\theta }_{0}^{{vm}}}$. Species names are ordered top_*-*_down according to their *R*_*θ*_ means and correspond in color to their respective crosses in **a** and **b**. Crosses with dotted lines in **a** represent *DISC* trials, which were subsampled to *N*_*obs*_ = 21; for these species, colors match the names in **b**. The species’ full names are provided in Table 1. Chi-square goodness of fit plots comparing the ${\hat{R}}_{\theta }$ quantiles distributions (black bars) to the null quantile distributions (grey bars) for *CRW-vm* (**c**) and *CRW-r* (**d**). **e** A heatmap of all trial counts binned at 5% increments according to their ${\hat{R}}_{\theta }$ quantiles in the *CRW-vm* and *CRW-r* analyses.

Out of 832 examined trials, 755 (91%) were indicated as directional (Rayleigh’s test, p < 0.05), and were thus considered for the quantitative analyses (Table 1). The results of the *Correlated Random Walk-von Mises* (*CRW-vm*) analysis (detailed in the Methods section) show that the observed mean vector length (${\hat{R}}_{\theta }$) of all tested species exceeded the mean expected under *CRW* (${\bar{R}}_{{\theta }_{0}^{{vm}}}$, Fig. 1a, b, Table 1). *DISC* trials carried out in the Mediterranean Sea (e.g., *Chromis chromis)*, exhibit generally lower ${\hat{R}}_{\varDelta \theta }$ compared to trials conducted in the Red Sea and Great Barrier Reef (e.g., *Chromis atripectoralis;* Fig. 1b, Table 1). Subsampled *DISC* trials exhibit similar quantile ranges to those of the *scuba-following* trials (dotted crosses in Fig. 1a), suggesting a solid comparability among the two sampling methods. In five species, confidence intervals overlap with ${\bar{R}}_{{\theta }_{0}^{{vm}}}$ (Fig. 1a, b, Table 1). Only two of these species, *Chaetodon aureofasciatus* and *Amblyglyphidodon curacao* do not display a clear indication for oriented movement, likely because of their distinctive depth-dependent behavior Supplementary note 1, Supplementary Figure S1. In the three other species (*Platax teira, Epinephelus coioides* and *Caranx ignobilis*), the small number of trials (2–15) per species may explain the confidence intervals overlap (Table 1).

Fourteen species have enough trials (*N*_*trials*_ ≥ 20) for a within-species chi-square test (proportions: 0–50%, 50–75%, and 75–100%). Of these, eleven species exhibit significant indication for oriented movement for both *CRW-vm* and *CRW*-*resampling* (*CRW-r*) analyses (chi-square test, *P* < 0.05, Cohen’s W > 0.5; Table 1). The chi-square test results support a significant indication for straighter movement than expected under CRW as the mean of effect sizes (Cohen’s W) is significantly larger than 0.5 for *CRW-vm* and *CRW-r* (One-tailed one-sample *t*-test, *t* > 1.93, *P* < 0.05).

*CRW-vm* and *CRW-r* quantile analyses provide a significant indication for straighter movement than expected under CRW because the ${\hat{R}}_{\theta }$ quantiles are significantly skewed towards higher values compared to the null (${R}_{{\theta }_{0}^{{vm}}}$ and ${R}_{{\theta }_{0}^{r}}$, Fig. 1c, d; chi-square test, *P* < 0.0001, Cohen’s W > 0.5). If larvae are using only internal cues for directional swimming, exhibiting *CRW* (i.e., unoriented movement), their density distribution should be centered around the medians (Fig. 1e, white circle). The high larval concentration on the top-right quarter of Fig. 1e provides a strong indication for straighter movement than expected under *CRW*, suggesting the use of external cues for directional movement by fish larvae (i.e., oriented movement).

In addition, *CRW- wrapped Cauchy* (*CRW-wc*) analysis–which includes a *wrapped Cauchy* fundamental distribution (instead of *von Mises*)–produces similar results to that of the *CRW-vm* analysis. Specifically, the means of all species fall above the ${\bar{R}}_{{\theta }_{0}^{{wc}}}$ curve, with mean quantiles higher than 50 (Supplementary note 2 and Supplementary Figure S2).

## Discussion

The set of quantitative analyses used in the study indicate a significantly straighter movement of larval fish than expected under CRW. These analyses are based on comparing the observed movement patterns to null distributions expected under CRW, produced by two complementary approaches: theoretical fundamental distribution (*CRW-vm* and *CRW-wc*) and resampling (*CRW-r*). This combined approach provides support for *oriented movement* by fish larvae.

**Fig. 2:**
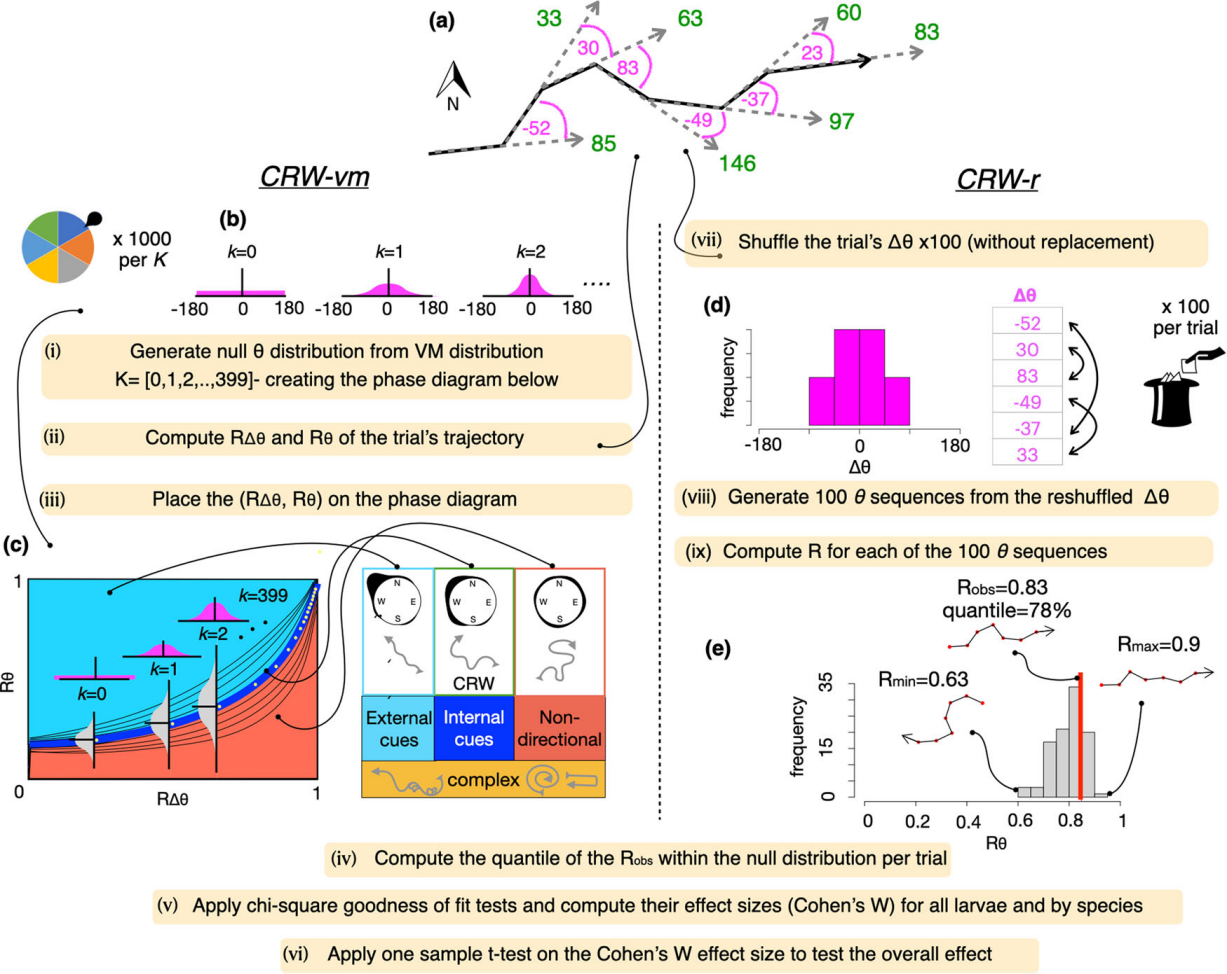
Protocol of the two quantitative methods applied in the study: Correlated Random Walk-von Mises (*CRW-vm*; left section), Sequence Resampling (*CRW-r*; right section). **a** Each trial includes a *bearings* sequence (*θ;* green), from which the *turning angles* sequence (*Δθ*; magenta) is computed. For visual clarity, **a** includes only seven bearings (*N*_*obs*_ = 7), while in the actual trials *N*_*obs*_ is larger. **b** (i) To generate the *CRW-vm* null distribution, we sample (with replacement) from a von Mises distribution, generating for each *kappa* value (*K*:0,1,2,3…, 399) 1000 *Δθ* sequences and corresponding *θ* sequences at the same length as *N*_*obs*_ per trial. (ii) For each of these simulated sequences, mean vector lengths *(R)* of *Δθ* and *θ* are computed (*R*_*Δθ*_, *R*_*θ*_), such that a distribution of *R*_*θ*_ (grey vertical distributions in **c**) is generated for each *kappa* (yellow dots). The 5th, 10th, 20th,…., 90th, and 95th quantiles of each of these *R*_*θ*_ distributions is computed, and contours connect these quantiles across the different *R*_*θ*_ distributions (black contours in **c**), with the mean represented as a thick blue line. Samples with high ${\hat{R}}_{\theta }$ compared to ${R}_{{\theta }_{0}^{{vm}}}$_,_ above and further than the blue line in the cyan colored area, represent a tendency for oriented movement. Complex movement patterns are not suitable for analysis using our methodology and can occur in the area below ${R}_{{\theta }_{0}^{{vm}}}$, e.g., one-sided bias, as well as in the area above ${R}_{{\theta }_{0}^{{vm}}}$, e.g., composite correlated random walks or bi-model turning angle distribution (zig zag) (for more details see Supplementary note 4). Note that the schematics in **c** represent the fact that the mean vector length (straightness) of the trajectory decreases from directed through CRW, to completely random (Simple Random Walk) or complex displacement-reducing movement patterns. (iii) (${\hat{R}}_{\varDelta \theta },$ ${\hat{R}}_{\theta }$*)* is computed per experimental trial and plotted on the phase diagram both individually and by species. (iv) Quantiles of the ${\hat{R}}_{\theta }$ within the ${R}_{{\theta }_{0}^{{vm}}}$ are computed using a 2-D interpolation. (v) Chi-square tests are applied to compare the ${\hat{R}}_{\theta }$ and the ${R}_{{\theta }_{0}^{{vm}}}$ distributions; and the effect size of the chi-square test (Cohen’s W) is computed. (vi) A one sample *t*-test is applied to examine the significance of the effect size (Cohen’s W > 0.5) across all species. Correlated Random Walk- resampling (*CRW-r*; right section). For each trial, Δ*θ* is computed (**a**), as shown in **d**, which represents a histogram of Δ*θ* from the example sequence Supplementary. Then, Δ*θ* sequence is sampled without replacement 100 times (vii), and 100 *θ* sequences are generated (viii)_._ Next, (**e**) *R* is computed for each of these 100 *θ* sequences (${R}_{{\theta }_{0}^{r}}$) (ix), and the quantile of the ${\hat{R}}_{\theta }$ within the ${R}_{{\theta }_{0}^{r}}$ distribution is calculated (iv), in this example quantile = 78%. Then, stages (iv–vi) are applied the same as for the *CRW-vm* method, ultimately computing the strength of the differences between the ${\hat{R}}_{\theta }$ and ${R}_{{\theta }_{0}^{r}}$ quantile distributions.

Previous work that quantitatively distinguished between oriented and unoriented movement, e.g., Correlated versus Biased Random Walk (CRW vs. BRW), have used: (i) step-length dynamics and their interactions with *bearings*^28^, (ii) displacement-related metrics for gauging oriented movement, e.g., net squared displacement^12,29^, or (iii) net displacement^6,28^. In contrast, we focus on *bearings*, constructing CRW-expected pattern. Specifically, we examine the movement dynamics based on the relationship between the *R*_*θ*_ and *R*_*Δθ*_. In our analyses, every larva is gauged against its own potential of autocorrelated directional swimming (CRW) and can be readily compared against other larvae from either the same or different species (Fig. 2). Note that our analyses do not distinguish between the different types of directed movement (i.e., BRW vs. BCRW). In addition, while previous methods determined indication for oriented movement if a given sequence exhibited orientation (e.g., displacement) above the 95% CI range expected for CRW, we employ a different quantitative approach in which we compare the observed distribution of trials’ quantiles to the distribution expected under CRW. The consideration of continuous quantiles (1–100%) rather than the dichotomous determination of within or outside the 95% CI range, increases the sensitivity of the analysis. Moreover, we provide a quantitative method for a meta-analysis of multiple individuals from multiple species that combines both theoretical and resampling approaches^6,12,28,30,31^.

Movement simulations often assume a von Mises or wrapped Cauchy distributions of *Δθ*, which are widely used to simulate CRW processes^6,11,13,22,32,33^. Yet, these distributions may not perfectly represent the true underlying distribution of *Δθ*. The strength of the *sequence resampling* (*CRW-r*) analysis is that it makes no assumptions regarding the distribution of *Δθ*, but instead reconstructs *θ* sequences by resampling the empirical *Δθ*^6,12,34^. Since these sequences are often short (*N*_*obs*_ = 21), they may not properly represent the true underlying distribution. Yet, the combination of resampling and theoretical distribution methods provides a complementary view and both support oriented movement in fish larvae. A subsampling of trials with high number of observations (*N*_*obs*_ = 180) produce comparable results to those of trials with a low number of observations (*N*_*obs*_ = 21) in terms of the range of quantiles for a given species (Supplementary note S3, Supplementary Figure S3).

Previous implementation of oriented (e.g., BRW)^22,35–38^ and unoriented (e.g., CRW)^22,32,39^ movement patterns in biophysical models of larval dispersal, demonstrated a significant effect on dispersal trajectories, settlement success and connectivity patterns^22,32,35–37^. The mathematical methodology behind such implementation has been extensively described^11^. It is largely based on a sampling of swimming directions from a von Mises or wrapped Cauchy distributions centered around the direction of the cue source for oriented movement, or around the swimming direction in the previous time step for autocorrelated movement^22,32,33^. In both cases, *kappa* or *rho*, the concentration parameters of the von Mises and the wrapped Cauchy distributions, respectively, govern the simulated precision of directional movement. Several studies used empirical species-specific data from in situ orientation trials to parametrize orientation behavior^22,36,40^. Yet, the combination and inter-dependence between oriented and autocorrelated directional movements were not implemented. The methodology presented here provides the basis for such implementation given the consideration of both oriented and unoriented movement under a single framework.

Animal orientation patterns are variable between species, between conspecifics, and within a single individual, with uncertainty and variability associated with environmental factors, availability of orientation cues, and the internal state of the organism^41^. In this work, we do not distinguish between higher order behaviors such as Biased Random Walk (BRW), Biased-Correlated Random Walk (BCRW), complex movement patterns such as chemotaxis^42,43^ or infotaxis^44^, and combinations of movement strategies^30,45,46^. We also disregard the spatial context of the movement trajectories and the possibility that 3-D movement may influence resultant 2-D orientation patterns^47^. Such complex behaviors are inappropriate for testing using our methodology. For example, animals that exhibit zig zag movement, with turning angles drawn from two distributions (centered around negative and positive values) or CRW movement sequences that are based of variable *kappa* could exhibit higher mean vector lengths compared to those expected under CRW (see Supplementary note S4, Supplementary Figure S4, and Supplementary Table S1 for more details). Other types of behaviors could be manifested in a relative reduction of ${\hat{R}}_{\theta }$ compared to what is expected given ${\hat{R}}_{\varDelta \theta }$, as observed in *C. aureofasciatus* and *A.curacao*. Hence, the interpretations provided in our study are based on the simplifying assumptions that a strict use of internal cues is expressed in a CRW process, and that oriented movement patterns result in more directional (or straighter) paths compared to unoriented patterns. We tested the possible effect of irregular behaviors on our results by removing trials that contained such irregular patterns and rerunning the analyses on this subset. The results still show a significantly straighter movement than expected under CRW, suggesting oriented movement by fish larvae (Supplementary note 5, Supplementary Figure S5).

In addition, spatiotemporal variability of the external cues should be considered and further examined in future studies. In our analyses we assumed that directional cues would come from a fixed direction throughout each trial. However, this assumption may not necessarily represent the conditions in the field. Part of the limitation lies in the fact that we cannot know for sure what are the directional cues utilized by fish larvae. For example, this assumption is correct if a larva was following a celestial cue^1,2^. In contrast, if a larva was following an auditory cue coming from a coral reef patch, and the larva was swimming or drifting, the direction of the cue source would change across the trial. However, considering the short durations of our trials (<15 min), currents and larval swimming speeds (<25 cm/s), the distance from shore in which the trials normally took place (>50 m), and the fact that potential sources of directional cues are often distributed parallel to the shore (e.g., fringing reef), we do not expect this to have a strong influence on the resultant movement properties of our trials.

Moreover, it is important to note that larvae in the field are often found in groups^22^, and larval swimming and orientation in groups are more efficient compared to single larvae^16,22^. In our results it seems that *Chromis atripectoralis* groups exhibited straighter paths and higher tendency for directed swimming compared to individual larvae based on both experimental methods. Yet, it is unknown if this effect is present in other species as well.

Previous studies indicated similarity in orientation patterns for *scuba-following* and *DISC* trials, with *scuba-following* exhibiting generally higher ${R}_{\theta }$ values^16,48^. The results summary in Table 1 suggests that *DISC* trials exhibit a higher tendency for oriented movement compared to the *scuba-following* trials. However, this is likely a result of the difference in the *N*_*obs*_. For *DISC* trials with low *N*_*obs*_, the ${\bar{R}}_{{\theta }_{0}^{{vm}}}$values are high, resulting in a more linear relationship, compared to trials with higher *N*_*obs*_ (Fig. 1a, b). Indeed, a subsampling of the *DISC* data to obtain *N*_*obs*_ = 21 results in a general reduction of *R*_*Δθ*_, *R*_*θ*,_ and the quantile of the trial. Notably, the *scuba-following* and subsampled *DISC* trials exhibit similar quantile ranges (Fig. 1a). In addition, it seems that *DISC* trials conducted in the Mediterranean Sea are characterized by lower *R*_*Δθ*_ compared to *DISC* trials from the Red Sea and the Great Barrier Reef. This might be related to differences in cue availability, that is affected by environmental conditions such as water turbidity and overcast sky conditions^19^. The fact that both the original and subsampled *DISC* datasets exhibited quantile distribution significantly higher than what is expected under CRW further supports the indication for directed movement by fish larvae.

Previous studies demonstrated that increase in sampling length (or reduction in sampling frequency) leads to an obstruction of the short-term persistence, making it appear more BRW, and leading to a decrease in the animal’s apparent speed^49,50^. Similarly, the computation of movement properties (path length, or the difference between observed and CRW-expected path length) given an incremental change in step-length shows that a directed movement is manifested especially across large scales^3,4^. In our data, due to the limited number of observations (*N*_*obs*_), such systematic analyses could not be applied. Future research should explore the effect of trial method, trial duration, sample frequency, and geographical location on the movement properties.

Overall, oriented movement allows a more persistent movement along a mean bearing over time compared to unoriented movement^6,13,26,28,32^. Such persistent movement results in an increased behavioral displacement relative to the water in which the larva swims, which in turn, modifies larval dispersal distance, recruitment success, and connectivity patterns^5,22,32,35,36^. Modifications are site- and species-specific, and are based on the fact that oriented movement allows the larvae to depart from entraining currents, and reach their settlement habitat more efficiently^22,32,35,37^. In our analyses, the high Cohen W effect size value (>0.5) suggests a high strength and consistency of oriented movement across individual larvae and pooled by species. It is important to consider intra-specific variation in orientation behavior, which may be a manifestation of bet hedging strategy^51^. Such variation should be further studied and implemented in biophysical models of larval dispersal.

Previous studies have demonstrated the need to consider larval orientation to understand the observed settlement success and connectivity^40^. It is therefore important to implement experimentally obtained orientation patterns into biophysical models, and the analytical approach presented here can increase the biological realism of larval dispersal models. Yet available orientation trials are of limited duration and longer trials are needed to study how orientation and its related cues change across time, space, and ontogeny. Future work should thus focus on isolating specific orientation cues, as well as studying cool-water, non-perciform taxa of commercial importance (e.g., Pleuronectiformes and Gadiformes) to promote a sustainable management of marine fish populations. Our meta-analysis suggests that fish larvae make substantial use of external cues for oriented movement, enabling them to find their way in the seemingly featureless, open ocean.

## Methods

### General methodological approach

To examine if larvae utilize external cues (i.e., oriented movement) to swim in a directional manner (i.e., significant mean vector length), we develop two complementary analyses that compare the empirically observed directional precision (i.e., mean vector length) with the null distribution expected under a strict use of internal cues (i.e., unoriented movement). The empirically observed directional precision is quantified as the mean vector length *(R)* of larval bearings (*θ)* (Fig. 2a), herein ${\hat{R}}_{\theta }$. The angular differences between consecutive bearings, herein *turning angles* (Fig. 2a; *Δθ*_*t*_ = *θ*_*t*_*-θ*_*t-1*_), are used to generate two null distributions of *R*_*θ*_ expected under the unoriented movement of *Correlated Random Walk (CRW;* ${R}_{{\theta }_{0}}$), based on the two analyses: *Correlated Random Walk-von Mises (CRW-vm)* and *Correlated Random Walk- resampling (CRW-r)*, described below. The first is theoretical and is based on a von Mises distribution of simulated *Δθ* (Fig. 2b, c); the second is empirical, and is based on resampling the *Δθ* within each trial (Fig. 2d, e). These two analyses are complementary because the first can generate an unlimited number of trajectories but is based on a theoretical distribution rather than on observations, whereas the second is based on a finite number of observations. In addition to these two main analyses, we apply a third analysis, the *Correlated Random Walk-wrapped Cauchy*, herein *CRW-wc*, which is similar to *CRW-vm*, with the only difference of using wrapped Cauchy distribution instead of von Mises. The reason for applying *CRW-wc* is that it was shown to represent well animal movement in some cases^33^. Notably, we consider the simple cases of undirected movement pattern with a turning angle distribution centered at 0 (CRW), testing if the mean vector length of the trial’s sequence is higher than that expected under CRW. If true, that would be an indication for a directed movement pattern (i.e., BRW or BCRW), or an indication for more complex behaviors (discussed in Supplementary note 4).

### Statistics and reproducibility

Quantitative analyses are applied to directional trials, i.e., larval *bearing* sequences ($\hat{\theta }$) that are significantly different from a uniform distribution based on the Rayleigh’s test^8^ (*p* < 0.05). We compute the quantiles in which the observed precision (${\hat{R}}_{\theta }$) of each trial falls within the null distributions (${R}_{{\theta }_{0}^{{vm}}}$and ${R}_{{\theta }_{0}^{r}}$, computation explained below), and compare these quantile distributions with the null quantile distributions using a chi-square test, gauging the observed directional precision ${\hat{R}}_{\theta }$ against the potential of autocorrelated precision (${R}_{{\theta }_{0}}$). We employ the simplifying assumptions that a strict use of internal cues is expressed in a CRW process, and that oriented movement patterns result in more directional (or straighter) paths compared to unoriented patterns. Under these assumptions we expect that the empirical ${\hat{R}}_{\theta }$ will exceed the autocorrelated pattern ${R}_{{\theta }_{0}}$ for individuals that apply oriented movement, whereas for an unorienting individual, ${\hat{R}}_{\theta }$ is expected to be closer to ${\bar{R}}_{{\theta }_{0}}$^6^ (Fig. 2c). Note however, that it is often difficult to distinguish between oriented and unoriented movement over a short duration (Fig. 2c); the differences between these two types of movements are much more apparent over long time duration, with oriented movement achieving greater displacement compared to unoriented movement^45^. ${\hat{R}}_{\theta }$ values less than ${R}_{{\theta }_{0}}$may result from complex behaviors such as one-sided bias (left or right), representing the utilization of internal cues (Fig. 2c, Supplementary Information section S1). In addition, our methodology is not appropriate when movement patterns are complex, e.g., CRW composite, in which there is more than a single CRW pattern per a given sequence, and zig zag patterns, in which consecutive turning angles are drawn from von mises distributions centered around positive and negative values, respectively (Supplementary note 4).

We apply our methods to a database of 835 in situ orientation trials gathered on larvae of 21 species from eight families of perciform fishes at various tropical and warm-water locations in East Asia^14,15^, Australia^16–18^, Mediterranean^52^ and Red Sea^19^, synthesized from eight previously published studies (Table 1). These studies examined the orientation behavior of settlement-stage^17^ and pre-settlement-stage^18^ larvae^15^ of reef-^14^, non-reef^20^, and pelagic^15^ fish species. In addition, some of these studies examine whether larval fish use directional information from the sun for oriented movement^19,52^, as well as the difference in orientation patterns between individuals versus groups of larvae^16^.

The methodology used for these in situ studies can be divided into two main categories. First, with direct observations through *Scuba-Following*, where a larva is released in the pelagic environment and tracked by scuba divers for 10 min, during which swimming direction is recorded every 30 s, resulting in 21 observations (*N*_*obs*_ = 21); for the full protocol, see^23,53^. Second, with observations using the Drifting In Situ Chamber (*DISC*^54^). For each *DISC* trial, a larva is placed into a circular chamber, and its position is recorded for 15–20 min with an upward-looking camera fixed *circa* 50 cm below the chamber. The first 3–5 minutes of each *DISC* trial are considered as acclimation time and are excluded from the analysis, whereas the residual 10–15 min are the actual observations used for the analysis.

The number of observations per *DISC* deployment (*N*_*obs*_ = 90, 180 or 300; see Table 1) varies with the recording frequency of larval positions, ranging from 2 to 10 seconds (Table 1). Some of the *DISC* trials had missing data due to the fact that the position of the larva is not always identified during the manual digitization of the *DISC* trials, due to the small size of the larva and due to unfavorable visibility conditions^54^. Trials that had *N*_*obs*_ smaller than the required number were not used for the analyses. For the *DISC* trials, *N*_*obs*_ that had to be larger than 90% of the maximal *N*_*obs*_ designated per group (i.e., *N*_*obs*_ > 81, 162, 270). Trials with *N*_*obs*_ higher than the maximal *N*_*obs*_ were trimmed to contain the maximal *N*_*obs*_ per species_,_ retaining the later-in-time data. For the scuba-following trials, the number of observations had to be *N*_*obs*_ > 20 due to the sensitivity of the analysis to a low number of observations. In other words, a low number of observations limits the capacity of the quantitative analyses to distinguish between oriented and unoriented movement patterns (see Supplementary note 3, Supplementary Figure S3). Importantly, both methods were shown to be robust in terms of artifacts and biases^55,56^, and have been tested together demonstrating high consistency in larval orientation results^16,48^.

Each orientation trial includes a sequence of larval swimming directions, termed *bearings (θ)* (Fig. 2a). For the *DISC* trials, *θ* are the cardinal directions of larval positions within the *DISC*’s chamber^55^. The angular differences between *θ* of consecutive time steps (*t*) are defined as *Δθ* (*Δθ*_*t*_ = *θ*_*t*_*-θ*_*t-1*_), such that for every *θ* sequence of a given length (*N*), there is a respective *Δθ* sequence of length *N-1* (Fig. 2a). Directional precision with respect to external and internal cues is computed as the mean vector length of *bearings* (*R*_*θ*_) and of *turning angles* (*R*_*Δθ*_), respectively^54^_._ Values of mean vector length (*R)* range from 0 to 1, with 0 indicating a uniform distribution of angles and 1 indicating that all angles are the same.

We used two quantitative approaches to examine if larvae exhibit oriented movement: the *Correlated Random Walk- von Mises* and *Correlated Random Walk- wrapped Cauchy* (*CRW-vm* and *CRW-wc*) analyses and the *CRW resampling (CRW-r)* analysis. Both types of analyses are based on the assumption that trajectories of animals that strictly use internal cues for directional movement are characterized by a *CRW* pattern. Hence, their capacity for directional movement is exclusively dependent on the distribution of their *turning angles (Δθ)*^57^. In contrast, for an external-cues orienting animal, for which movement directions are correlated with an external fixed direction, the mean vector length of the observed *bearings*, ${\hat{R}}_{\theta }$, is expected to exceed that of a *CRW*, ${R}_{{\theta }_{0}}$^6^. Both analyses compare ${\hat{R}}_{\theta }$ against the expected ${R}_{{\theta }_{0}}$, but the first type computes ${R}_{{\theta }_{0}^{{vm}}}$and ${R}_{{\theta }_{0}^{{wc}}}$using theoretical von Mises and wrapped Cauchy distributions of *Δθ*, and the second type computes ${R}_{{\theta }_{0}^{r}}$ by producing 100 new *θ* sequences per individual trial (larva) by multiple resampling-without-replacement of the *Δθ*.

A key principle for both analyses types stems from the fact that the mean vector length of *bearings* (*R*_*θ*_) is inherently dependent on the mean vector length of *turning angles* (*R*_*Δθ*_)^28^. In other words, an animal with a high capacity for unoriented directional movement, i.e., a narrow distribution of *Δθ*, is likely to yield a high *R*_*θ*_, even if it makes absolutely no use of external cues for oriented movement. Hence, in both analyses ${\hat{R}}_{\theta }$ is gauged against a distribution of ${R}_{{\theta }_{0}}$, given its respective mean vector length of *turning angles* ${\hat{R}}_{\triangle \theta }$. The open-source software *R*^58^ with the package circular^59^ is used for all analyses in this study.

### Correlated Random Walk-von Mises (CRW-vm)

In this analysis, we first generate the directional precision (*R*), expected for unoriented CRW movement using the theoretical von Mises distribution (${R}_{{\theta }_{0}^{{vm}}}$). The CRW *bearings* sequences (${\theta }_{0}^{{vm}}$) are generated by choosing a random initial bearing, followed by a series of *N*_*obs*_*-1 turning angles (*${\triangle \theta }_{0}^{{vm}}$) in *bearing* direction; drawn at random (with replacement) from a von Mises distribution (*N*_*rep*_ = *1000*). The length of ${\theta }_{0}^{{vm}}$ sequence is according to the number of observations in our four types of experimental trials: *N*_*obs*_ = 21 for the *scuba-following*, and 90, 180 and 300 for the *DISC* (Table 1). The directional precision of the von Mises distribution is dependent on the concentration parameter, *kappa*. *Kappa* values ranging from 0 to 399 are applied at 1-unit increments to cover the entire range of directional precision from completely random (*kappa* = 0), to highly directional (*kappa* = 399). Next, the directional precision of the *bearings* (*R*_*θ*_) and the *turning angles* (*R*_*Δθ*_) are computed for each simulated sequence of *θ* (Fig. 2a–c).

These respective pairs of values (*R*_*Δθ*_*, R*_*θ*_) provide the basis for generating the expected relationship between ${R}_{{\theta }_{0}^{{vm}}}$ and ${R}_{{\triangle \theta }_{0}^{{vm}}}$. Then, for any given *kappa* value, the following quantiles are computed: 5th, 10th, 20th,….,90th, and 95th (grey vertical distributions in Fig. 2c). Next, smooth spline functions are fitted through all respective quantiles, generating the ${R}_{{\theta }_{0}^{{vm}}}$quantile contours, which represent the null expectation under CRW. This expected (*R*_*Δθ*_, *R*_*θ*_) correspondence creates a phase diagram (Fig. 2c), based on which the observed *θ* patterns are gauged. The procedure is repeated four times to match the among-study differences in the number of *θ* observations per trial (i.e., *N*_*obs*_ = 21, 90, 180, and 300; see Table 1).

To examine if the observed larval movement patterns differ from those expected for unoriented movement (*CRW-vm)*, we compute *R*_*Δθ*_ and *R*_*θ*_ for each individual trial (${\hat{R}}_{\triangle \theta }$ and ${\hat{R}}_{\theta }$). We then place these values in the phase diagram and examine their positions with respect to ${R}_{{\theta }_{0}^{{vm}}}$ (Fig. 2c). Larvae with ${\hat{R}}_{\theta }$ substantially higher than ${\bar{R}}_{{\theta }_{0}^{{vm}}}$, are considered to have a higher tendency for a straighter movement than expected under CRW, suggesting oriented movement such as BRW and BCRW (Fig. 2b, c)^6,28^. Larvae with ${\hat{R}}_{\theta }$ values substantially below ${\bar{R}}_{{\theta }_{0}^{{vm}}}$indicate irregular patterns such as a one-sided drift (right or left). A larva is considered directional if the *bearing* sequence ($\hat{\theta }$) is significantly different from a uniform distribution based on the Rayleigh’s test (*p* < 0.05)^8^. Non-directional larvae are characterized by low ${\hat{R}}_{\triangle \theta }$ and ${\hat{R}}_{\theta }$, and thus will be situated at the bottom left area in the phase diagram (Fig. 2c). 95% confidence interval (CI) was computed for each species’ trials (${\hat{R}}_{\triangle \theta }$, ${\hat{R}}_{\theta }$). The difference (*∆R*) between ${\hat{R}}_{\theta }$ and ${\bar{R}}_{{\theta }_{0}^{{vm}}}$ was computed per each trial and pooled by species to assess the tendency for a straight movement compared to that expected under CRW, as an indication for oriented movement.

The quantile (*Q*) of each trial is then computed based on the location of (${\hat{R}}_{\triangle \theta }$, ${\hat{R}}_{\theta }$) within the null quantiles’ contours in the phase diagram (Fig. 2c), using a 2-D interpolation such that *X* = *R*_*Δθ*_, *Y* = *R*_*θ*_, and *Z* = *Q* (Akima R package^60^; Fig. 1b). 2-D interpolation is used once more to overlay the (${\hat{R}}_{\triangle \theta }$, ${\hat{R}}_{\theta }$) of two species with a different number of observations (*N*_*obs*_ = 300: *Premnas biaculeatus, N*_*obs*_ = 90 *Chromis atripectoralis*) on the *DISC*’s phase diagram (*N*_*obs*_ = 180), which represents most of the *DISC* trials.

### Correlated Random Walk-wrapped Cauchy (CRW-wc)

Although von-Mises distribution is the most commonly used circular distribution for simulating CRW^11^, the wrapped Cauchy distribution well represents the underlying distributions in some cases^33^. To examine the sensitivity to the underlying distribution of our method, we repeated the exact same protocol of *CRW-vm*, only with a wrapped Cauchy distribution instead of von-Mises, and respectively, using the *rho* concentration parameter instead of *kappa*, with values (*n* = 400) ranging between 0 and 0.999, representing rho’s minimum and maximum values.

### Correlated Random Walk- resampling (CRW-r)

In this analysis, we generate ${R}_{{\theta }_{0}}$ expected under a strict use of internal cues of *CRW* pattern using resampling of the *turning angles (Δθ)* per trial sequence (i.e., ${R}_{{\theta }_{0}^{r}}$). Specifically, for every trial sequence, ${R}_{{\theta }_{0}^{r}}$ is computed by generating 100 *θ* sequences from 100 resampled *Δθ* sequences (*N*_*rep*_ = *100*, without replacement) from the empirical *Δθ* (Fig. 2d). The ${R}_{{\theta }_{0}^{r}}$ sequence length is equal to the number of observations in each trial (*N*_*obs*_). Next, *R*_*θ*_ for each of the resampled sequences (${R}_{{\theta }_{0}^{r}}$) and the quantile in which ${\hat{R}}_{\theta }$ falls within ${R}_{{\theta }_{0}^{r}}$, are computed (Fig. 2e). The quantile represents the proportion of ${R}_{{\theta }_{0}^{r}}$ which is smaller than ${\hat{R}}_{\theta }$ for each trial.

### Meta-analysis chi-square goodness-of-fit tests

To test if ${\hat{R}}_{\theta }$ was significantly higher than what is expected under the null, we used chi-square tests to compare the ${\hat{R}}_{\theta }$ quantile distributions (observed trial counts) with the null (${R}_{{\theta }_{0}^{{vm}},}\,{R}_{{\theta }_{0}^{{wc}}}$and ${R}_{{\theta }_{0}^{r}}$) quantile distributions (simulated sequences counts). For applying chi-square tests on larvae of different species pooled together, we used the following quantile partitioning: 0–50%, 51–70%, 71–90%, and 91–100%. For applying chi-square tests on larvae pooled by species, we used the following quantile partitioning: 0–50%, 51–75%, 76–100%. The reason for the differences is the minimal number of samples limitation of the chi-square goodness-of-fit test, which is a minimum of 5 samples per expected bin^61^. This limitation allows a minimum of 20 samples in the species chi-square test, and 50 samples in the chi-square test for all larvae pooled together. Importantly, the analysis is done on counts of the individual trials’ values, thus there is no information loss due to pre-analysis pooling of data.

To test whether ${\hat{R}}_{\theta }$ are significantly higher than what is expected under the null across species, we computed the effect size (Cohen’s W) of each chi-square test per species, and examined if the effect sizes distribution is significantly higher than 0.5 using a one-sided one-sample t-test, after ensuring normality of effect sizes distribution using Shapiro-Wilk test^62^. Effect size of Cohen’s W ≥ 0.5 represents a strong effect size for the chi-square goodness-of-fit test^63^. This analysis was applied to trials that contained single larva rather than groups, as grouped larvae were shown to orient differently than single larvae^16^.

To examine the correspondence between the ${\hat{R}}_{\theta }$ quantiles of the two methods: *CRW-vm* and *CRW-r*, the quantiles (*Q*) data were binned at increments of 5% for the two analyses, creating a 20 × 20 cell matrix (*M*). Then, the matrix was filled based on the ${\hat{R}}_{\theta }$ quantiles of the larvae, such that for example, a given larva with *Q*_*CRW-vm*_ = 99% and *Q*_*CRW-r*_ = 96%, will be counted in *M*_*20,20*._ Whereas, a given larva with *Q*_*CRW-vm*_ = 52% and *Q*_*CRW-r*_ = 46%, will be counted in *M*_*11,10*_. Based on this matrix, the corresponding heatmap contoured plot in Fig. 1e was produced, using the R package plot3D^64^.

If the two methods of analysis (*CRW-vm* and *CRW-r*) provide significant test results for a given species, this can be regarded as evidence for oriented movement under our simplifying assumptions. If both methods fail to reject the unoriented movement null hypothesis, it seems likely that external cues are not used for directional movement. However, if the two methods provide differing test results, no definitive conclusion about how directional movement is maintained can be reached.

Subsampling of the *DISC* trials was carried out, obtaining subsampled sequences with *N*_*obs*_ = *21*. This was done to examine the effect of variation in *N*_*obs*_ on the analyses results (i.e., quantiles and *△R*_*θ*_), and to compare between the *DISC* and the *scuba-following* trials under a similar *N*_*obs*_. Subsampled trials underwent the same filtering scheme as the regular trials in terms of the mean vector significance (Rayleigh’s test) and the available number of observations.

### Reporting summary

Further information on research design is available in the Nature Research Reporting Summary linked to this article.

## Supplementary information

Supplementary Information

Description of Additional Supplementary Files

Supplementary Data 1

Supplementary Code 1

Reporting Summary

## Data Availability

The data that support the findings of this study are available from the corresponding author upon reasonable request. Sample data that includes bearings sequences of *Caesio cuning* trials is provided in supplementary data 1.

## Code availability

Data were analyzed using R version 4.0.3. R code that includes the analyses of this study is provided in supplementary code 1.
