# Supplementary material for: Evidence for a consistent use of external cues by marine fish larvae for orientation
Source: Commun Biol. 2022 Dec 2;5:1307. doi: 10.1038/s42003-022-04137-7 (PMC9718780; doi:10.1038/s42003-022-04137-7)
Supplement: Supplementary file 1 — Supplementary Information [file 42003_2022_4137_MOESM1_ESM.pdf]

Supplementary information

Evidence for a consistent use of external cues by marine fish larvae for orientation

Igal Berenshtein[1,2,3], Robin Faillettaz[1,4,5], Jean-Oliver Irisson[4], Moshe Kiflawi[6,7], Ulrike E. Siebeck[8], Jeffery M. Leis[9,10], Claire B. Paris[1*]

[1]*Rosenstiel School of Marine and Atmospheric Science University of Miami 4600 Rickenbacker Causeway, Miami, Florida 33149, United-States*
[2]*Cooperative Institute for Marine and Atmospheric Studies, Rosenstiel School of Marine and Atmospheric Science, University of Miami, 4600 Rickenbacker Causeway, Miami, FL 33149, USA*
[3] *Department of Marine Biology, Leon H. Charney School of Marine Sciences, University of Haifa, Mt. Carmel 3498838, Haifa, Israel*
[4]*Centre National de la Recherche Scientifique, Laboratoire d'Océanographie de Villefranche-sur-Mer (LOV), Sorbonne Universités, UPMC University Paris 06, Villefranche-sur-Mer, France*
[5]*EDECOD (Ecosystem Dynamics and Sustainability), IFREMER, INRAE, Institut Agro, Lorient, France*
[6]*Department of Life-Sciences, Ben-Gurion University of the Negev, POB 653, 84105 Beer-Sheva, Israel*
[7]*The Interuniversity Institute for Marine Sciences of Eilat, Eilat, 88103, Israel*
[8]*Laboratory for Visual Neuroethology, School of Biomedical Sciences, University of Queensland, St Lucia, QLD 4072, Australia*
[9]*Ecology and Biodiversity Centre, Institute for Marine and Antarctic Studies, University of Tasmania, Hobart, TAS, 7007, Australia*
[10]*Ichthyology, Australian Museum Research Institute, Sydney, NSW, 2001, Australia*

[*]Corresponding author: cparis@earth.miami.edu

Supplementary Note 1. Movement analysis of species which do not exhibit higher mean vector lengths than expected under CRW

According to the *CRW-vm* simulation analysis, *Chromis atripectoralis*, *C. aureofasciatus* and *A. curacao* do not seem to do not exhibit a significantly straighter movement (i.e., higher mean vector length) than expected under CRW, as their *CRW-vm* quantile distribution was not significantly different from the null (i.e., P>0.05, Cohen's W<0.25, Figure S1, Table 1). In contrast, the *CRW-r* analysis of these species indicated that they indeed exhibit a significantly straighter movement than expected under CRW, yet their effect sizes were the lowest among all species (Table 1). Further details regarding the dynamics of *scuba-following* trials [1–3] indicate that multiple individuals from *C. aureofasciatus* and *A. curacao* were characterized by distinctive behavior of immediate descent after their release by the observing divers (Figure 2 in [1] and Figure 6 in [3]). This behavior possibly alters the natural orientation behavior observed in the other species (Figure 1), resulting in multiple individuals with $\hat{R}_\theta$ values below their expected $R_{\theta_0^{vm}}$ range (Figure S1). Such pattern can be a result of complex behaviors such as a consistent drift to either right or left, which is manifested as an asymmetrical distribution of *turning angles* (*$\Delta\theta$),* that is not centered around zero (Figure 1c, Figure S1).

For example, the *C. aureofasciatus* larva marked in Figure S1a is characterized by a narrow and asymmetrical distribution of $\Delta\theta$ (Figure S1b), and a nearly uniform distribution of θ (Figure S1c). The dynamics of θ across time (Figure S1c) indicate that the larva consistently made small turns to the left, completing a nearly full circle. Therefore, it is not surprising that $\hat{R}_\theta$ is lower than expected under $R_{\theta_0^{vm}}$, as the larva is essentially swimming in a circle. Similarly, the $\hat{R}_\theta$ quantiles of that specific larva is 3% and 1% for the *CRW-r* analysis and the *CRW-vm* analyses respectively, supporting the same conclusion.

A further factor for *A. curacao* is that changes in θ between successive measurements ($\Delta\theta$) were biased to the left (57.8% left and 42.2% to right), but also differed in the size of the bias and size of $\Delta\theta$ depending on the swimming direction[3], i.e., more left than right turns, or made larger left turns, or both. Interestingly, for a larva swimming to the northwest (270° to 360°), left $\Delta\theta$ were 1.67 times more likely, but also the average size of a left $\Delta\theta$ was 6° greater than a right $\Delta\theta$. In contrast, for larvae swimming to the southeast (90° to 180°), a left $\Delta\theta$ was 1.4 more likely than a right $\Delta\theta$, but was only 2° larger. As a result, a larva swimming to the southeast would change direction less over time than a larva swimming in other directions, and those other larvae would be more likely to eventually turn around towards the southeast and southwest.

Supplementary Note 2. Wrapped Cauchy as an underlying distribution

Although von Mises distribution is the most commonly used circular distribution for simulating Correlated Random Walk (CRW), other distributions such as wrapped Cauchy were suggested to be more suitable in some cases, for example, when the underlying distribution is a combination of two or more distributions[4]. Based on this we tested the sensitivity of the *CRW-vm* analysis to the underlying type of distribution by replacing the von Mises distribution with a wrapped Cauchy distribution (*CRW-wc*), and accordingly, replacing the *kappa* concertation parameter (n=400, ranging from 0 to 399) with *rho* concertation parameter (n=400, ranging from 0 to 0.999). The results indicate that there was no major difference evident between *CRW-vm* and *CRW-wc,* except for the fact that the distribution of quantiles was wider towards the extreme values of $\Delta\theta$ in the x-axis (i.e., $\Delta\theta$=0 and $\Delta\theta$=1). For the *CRW-wc* analysis, same as for *CRW-vm* analysis, the means of all species fall above the $\bar{R}_{\theta_0^{wc}}$ curve, the confidence intervals of the bulk of species do not overlap with the $\bar{R}_{\theta_0^{wc}}$ (Figure S2).

Supplementary Note 3. Comparison between scuba-following and *DISC* experiments for *Chromis atripectoralis*

Previous studies examined the differences in the orientation patterns of *C. atripectoralis* between *scuba-following* and *DISC* experiments and between individuals and groups of larvae[5,6]. These studies showed consistency in the findings between the methods, namely in the median swimming directions, the level of directional precision (R_θ). The studies indicated that while both experimental methods indicated that the bulk of larvae swim directionally (Rayleigh's test $p<0.05$), the *scuba-following* experiments were characterized by higher R_θ values. In addition, the experiments with groups of larvae were characterized by higher R_θ values in both methods. These findings are indeed evident in Figure S3, indicating higher R_θ values for the group experiments in both experimental methods, and higher R_θ values of *scuba-following* compared to the *DISC* experiments. The methodology provided here indicates a higher tendency of the *DISC* experiments for exhibiting a straighter movement than expected under CRW (i.e., higher $\triangle R_\theta$ and higher quantiles) compared to the *scuba-following*. A sub-sampling of the *DISC* experiments (to N_{obs} =21) yields comparable results, such that the quantiles of both methods, for individuals and groups, exhibits positive $\triangle R_\theta$ values, and quantile means that range between the 50th and the 85th quantiles, indicating a tendency for exhibiting a straighter movement than expected under CRW (Table 1).

The significance of the chi-square tests, however, is not consistent across the different sets of trials. For example, for the *DISC* trials, chi-square tests of *C. atripectoralis* individuals of both *CRW-vm* and *CRW-r* methods, are significant, but for the *C. atripectoralis* groups, *CRW-r* chi-square test is significant and *CRW-vm* is not (Table 1). For the *scuba-following* trails and for the subsampled *DISC* data, chi-square test was significant only for the *CRW-r* method for *C. atripectoralis scuba-following* trials. Chi-square test was not significant for *C. atripectoralis*

group subsampled *DISC* experiments or for the individual *C. atripectoralis CRW-vm* method of the *scuba-following* trials (Table 1). For the group *scuba-following trials*, there were not sufficient number of trials to conduct the chi-square goodness of fit analysis (Table 1).

Supplementary Note 4. Simulation of irregular unoriented movement patterns.

While our results provide indication for oriented movement by fish larvae, it does not cover all possible behaviors that may be exhibited by moving animals. Complex movement patterns that do not follow the classic definition of CRW, BRW and BCRW are not suitable for our analyses. In this section we simulate six different behaviors under variable number of observations and *kappa* values. Three of these behaviors are the classic patterns of CRW (unoriented), BRW and BCRW (oriented). The other three behaviors represent special patterns that include composite random walk (i.e., *kappa* changes during the trial), “zig zag” movement (i.e., $\Delta\theta$ are drawn from two von-Mises distributions centered around positive and one negative values), and one-sided drift (i.e., $\Delta\theta$ are drawn from a von-Mises distributions that is not centered around zero). While the classic patterns are located in the phase diagram as expected, with CRW on the $R_{\theta_0^{vm}}$ mean curve, BRW located above the 95% $R_{\theta_0^{vm}}$ quantile, and BCRW located in between the two. The special behaviors can be falsely categorized, for example the undirected “zig zag” and the composite CRW behaviors are categorized as indication for directed movement. Details about the simulations and the implemented movement parameters are given in Table S1.

Supplementary Note 5. Running our analyses on trials that do not exhibit irregular behavior.

In this section, we examine if trials with irregular behavior may affect our general results which indicate the use of external cues by fish larvae. To achieve this, we visually inspected the movement patterns of *scuba-following* and subsampled *DISC* trials (Nobs=21), and ran our

analyses on trials that do not seem, based on visual inspection, to demonstrate irregular behavior (i.e., one-sided bias, multi-modal distribution of θ, bi-modal distribution of $\Delta\theta$, composite movement pattern) (Fig. S5). An example of the plots used for the visual inspection are given in Figure S6 for a trial that does not demonstrate irregular behaviors, and in Figure S7 for a trial that demonstrates irregular behavior. This Supplementary note indicated that trials that may contain irregular behaviors do not affect the overall results and conclusion. It is important to note that it is difficult to differentiate between the possible types of irregular behaviors. Some trials represent a combination of a few types (e.g., one-sided drift and composite pattern), and it is also possible that there are other types of irregular behaviors that are not considered. In addition, it is possible that despite the irregular behaviors the trial would be correctly categorized, meaning that a larva that was actually using extremal cues for orientation would have been correctly categorized as such, and vice-versa.

Supplementary References

1. Leis, J. M. Vertical distribution behaviour and its spatial variation in late-stage larvae of coral-reef fishes during the day. *Mar. Freshw. Behav. Physiol.* **37**, 65–88 (2004).
2. Leis, J. M. & Carson-Ewart, B. M. Orientation of pelagic larvae of coral-reef fishes in the ocean. *Mar. Ecol. Prog. Ser.* **252**, 239–253 (2003).
3. Leis, J. M., Wright, K. J. & Johnson, R. N. Behaviour that influences dispersal and connectivity in the small, young larvae of a reef fish. *Mar. Biol.* **153**, 103–117 (2007).
4. Bailey, J. D. & Codling, E. A. Emergence of the wrapped Cauchy distribution in mixed directional data. *AStA Adv. Stat. Anal.* 1–18 (2020).
5. Irisson, J.-O., Paris, C. B., Leis, J. M. & Yerman, M. N. With a little help from my friends: group orientation by larvae of a coral reef fish. *PLoS One* **10**, e0144060 (2015).
6. Leis, J. M., Paris, C. B., Irisson, J.-O., Yerman, M. N. & Siebeck, U. E. Orientation of fish larvae in situ is consistent among locations, years and methods, but varies with time of day. *Mar. Ecol. Prog. Ser.* **505**, 193–208 (2014).

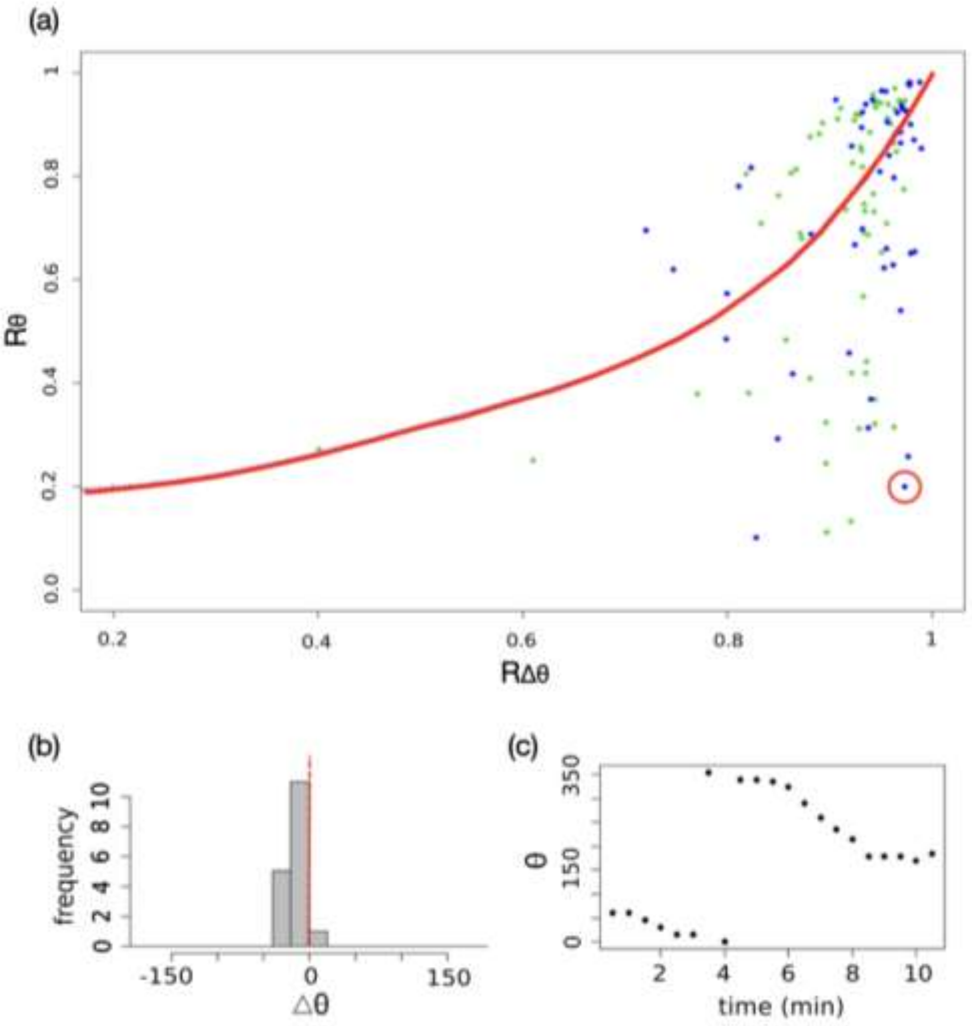

Figure S1. Example of Correlated Random Walk von Mises (*CRW-vm*) analysis of individual larvae of the species *Chaetodon aureofasciatus* and *Amblyglyphidodon curacao*. (a) $(\hat{R}_{\Delta\theta}, \hat{R}_{\theta})$ of *C. aureofasciatus* (blue) and *A. curacao* (green). Circled in red is an orientation trial of an individual *C. aureofasciatus* which did not exhibit a straighter movement (i.e., higher mean vector length) than expected under CRW (i.e., $\hat{R}_{\theta} < \bar{R}_{\theta_0^{vm}}$). Red line in (a) represents the mean *CRW-vm* (Figure 2). (b) *Turning angles* distribution ($\Delta\theta$), and (c) *bearings (θ)* versus trial time of the *C. aureofasciatus* larva marked in red in (a).

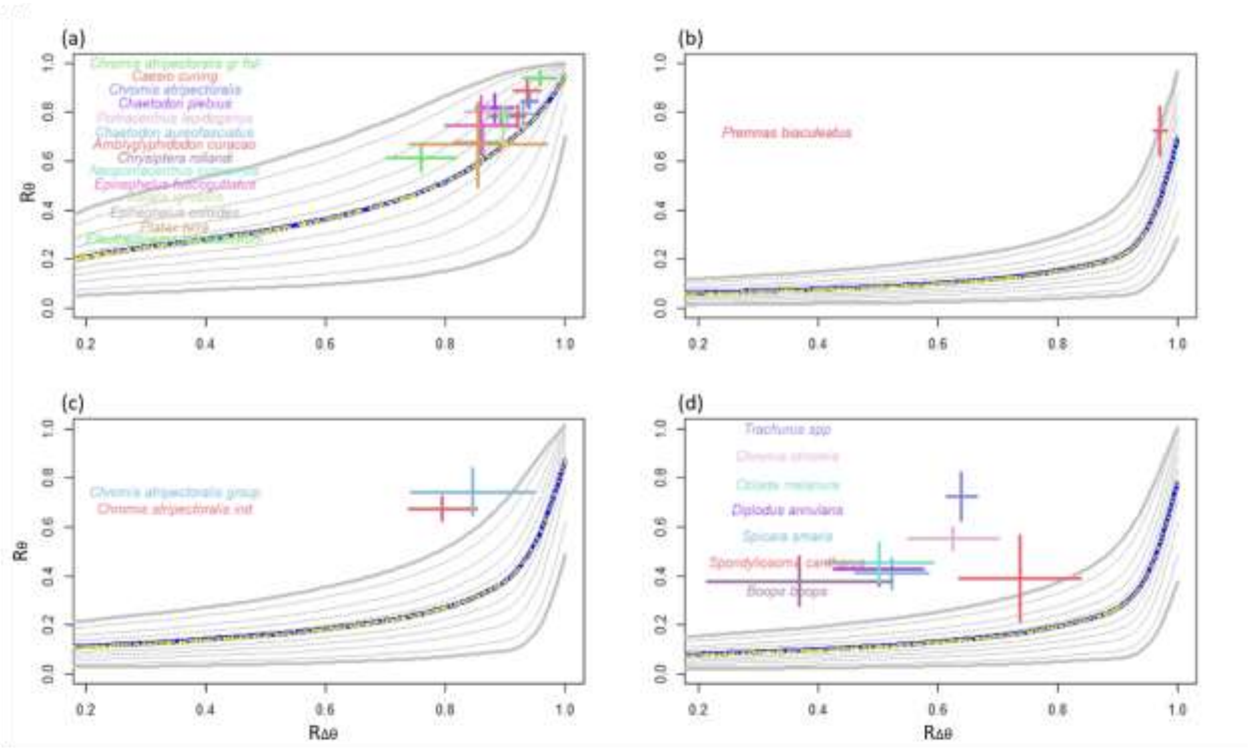

Figure S2. Correlated Random Walk wrapped Cauchy (*CRW-wc*) analysis at the species level. The analysis is based on the diagram in (Figure 2c) with the wrapped Cauchy distribution replacing the von Mises distribution. Panels represent the scuba-following trials (a), and for the *DISC* trials (b-d) with various number of samples-per-trial: *Nobs*=21 (a), 300 (B), 90 (c), and 180 (d). Crosses represent means ± 95% Confidence Interval (CI) of the observed ($\hat{R}_{\Delta\theta}$, $\hat{R}_{\theta}$) pooled by species. Thick blue line represents the curve fitted through the means of ($R_{\Delta\theta_0^{wc}}, R_{\theta_0^{wc}}$), generated for each *rho* value (yellow dots on top of the curve). Species names are ordered top-down according to their $\hat{R}_{\theta}$ means and correspond in color to their respective crosses.

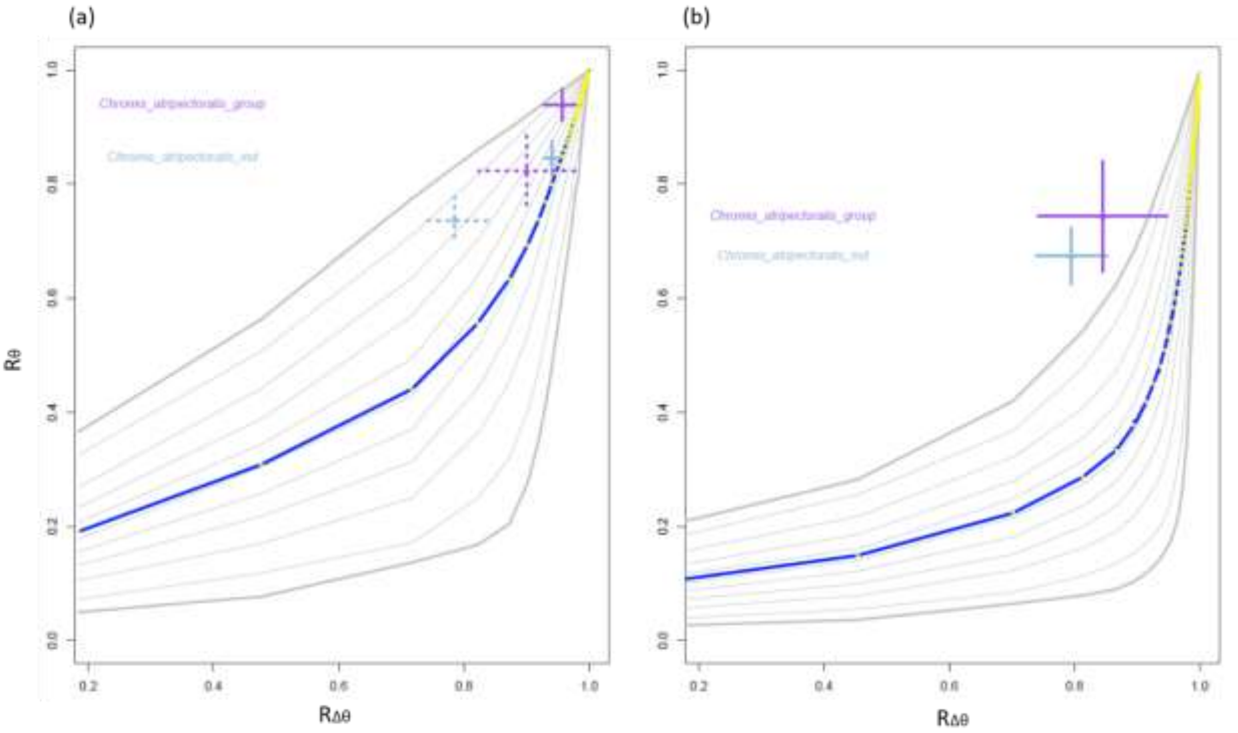

Figure S3. Comparison between scuba-following (a) and *DISC* (b) trials of individuals (cyan) and groups (magenta) of *Chromis atripectoralis* settlement-stage larvae using Correlated Random Walk von Mises (*CRW-vm*). Dotted crosses represent the trials in (b) which were sub-sampled to contain 21 observations (N_{obs}) per trial. The analysis is based on the diagram in (Figure 2c). Crosses represent means ± 95% Confidence Interval (CI) of the observed $(\hat{R}_{\Delta\theta}, \hat{R}_{\theta})$.

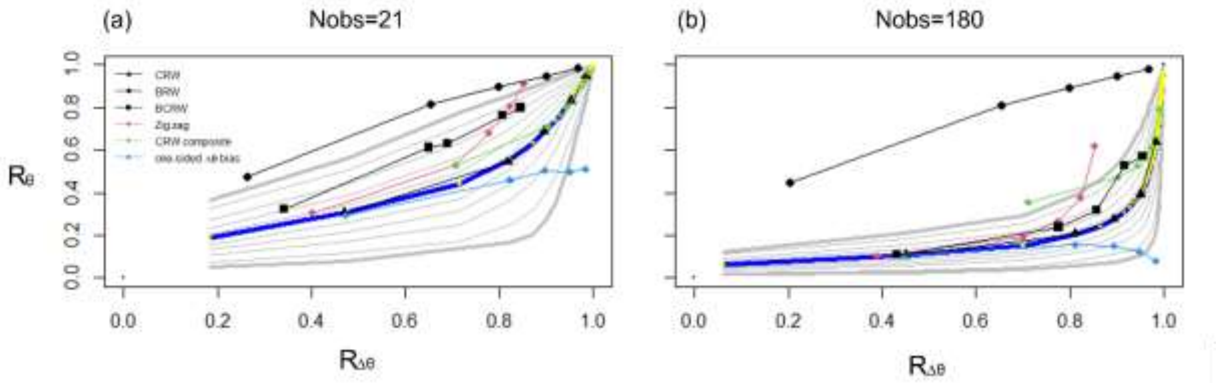

Figure S4. Simulated examples of classic and irregular patterns. Simulated classic patterns include Correlated Random Walk (CRW), Biased Random Walk (BRW), and Biased Correlated Random Walk (BCRW); irregular patterns include bimodal distribution on turning angles ($\Delta\theta$; zig zag), movement pattern in which *kappa* parameter of $\Delta\theta$ distribution changes halfway through the simulation (CRW composite), and one-sided $\Delta\theta$ bias. Movement patterns are simulated for *kappa*=1, 3, 5, 10, 30 and number of observations of (a) Nobs=21 and (b) Nobs=180. More details about theses simulations and their parametrization are given in Table S1.

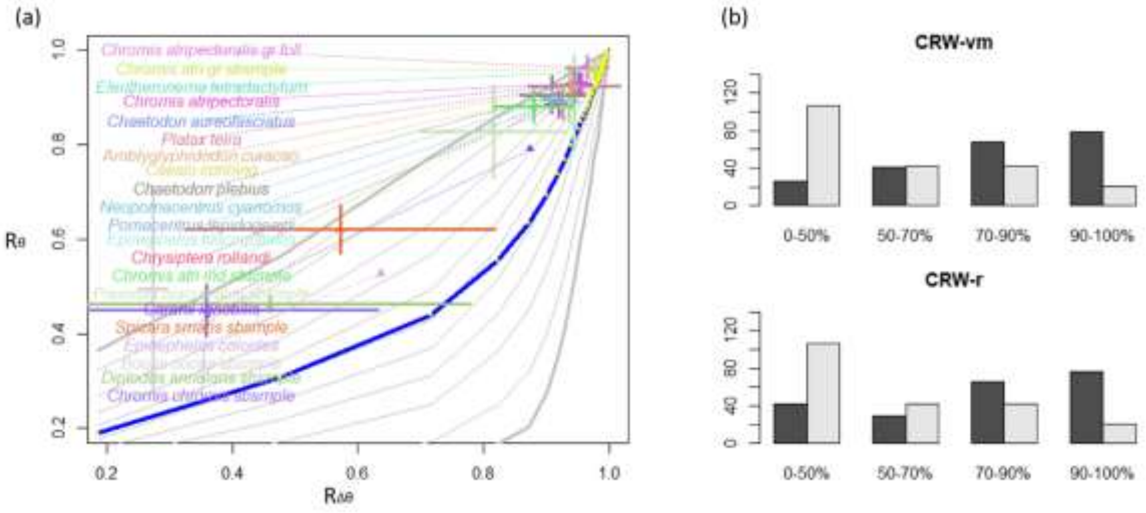

Figure S5. Correlated Random Walk-von Mises (CRW-vm) and Correlated Random Walk resampling (CRW-r) results for trials that do not demonstrate irregular behavior. This analysis was performed on trials with Nobs=21, i.e., on *scuba-following* and subsampled *DISC* trials that do not seem to demonstrate irregular behavior, i.e., one-sided bias, multi-modal distribution of bearings (θ), bi-modal distribution of turning angles ($\Delta\theta$), and *CRW* composite movement pattern (N=214,). Legend details are the same as in Fig. 1. Examples of trials without and with irregular patterns are given in supplementary Figs. S6 and S7, respectively. Triangles represent species that included a single individual in this analysis.

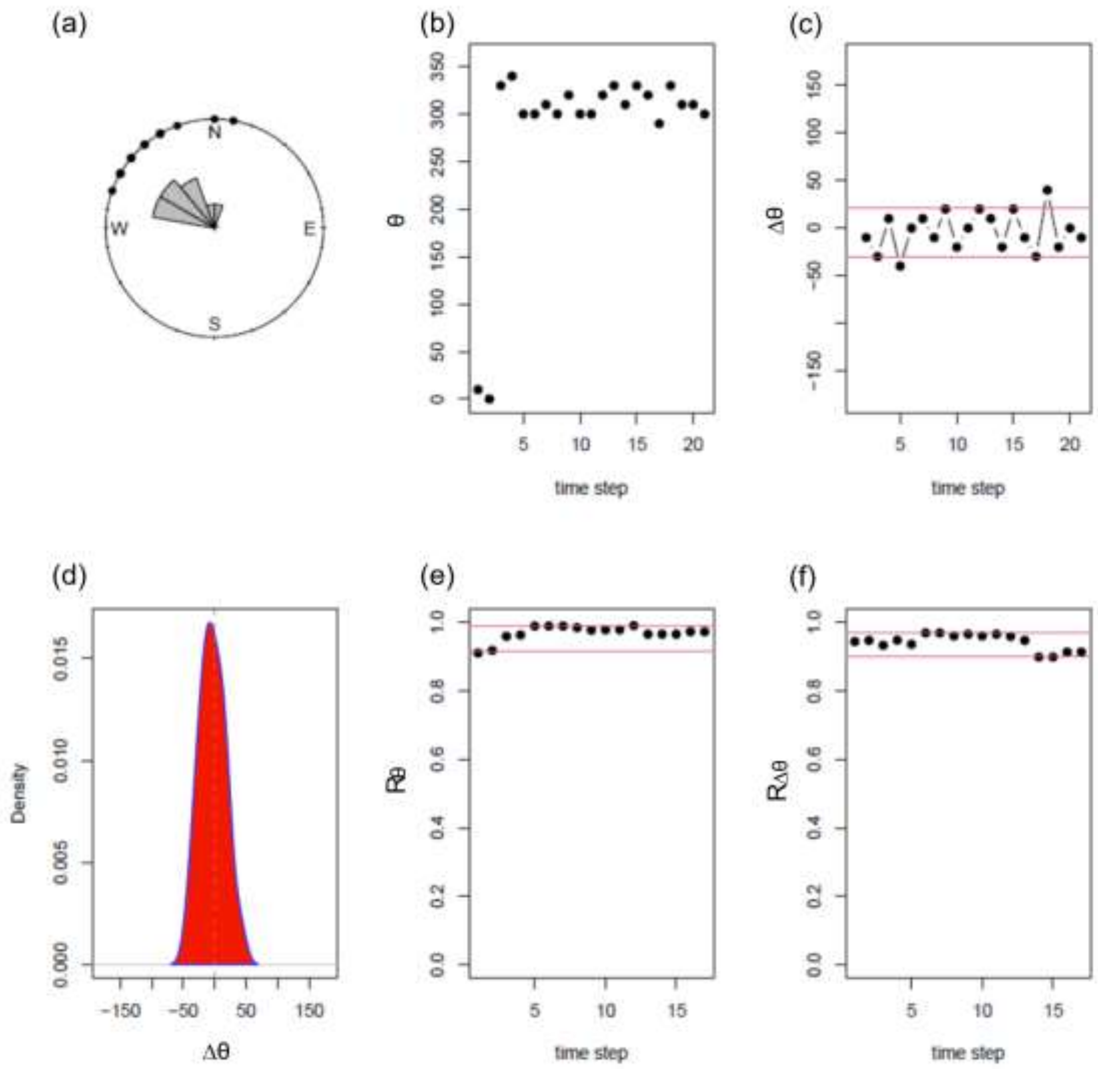

Figure S6. Visual inspection for detecting irregular behaviors- an example that does not show irregular patterns. An example a trial (*Caesio cuning, scuba-following*) that does not show irregular patterns of: one-sided bias, multi-modal distribution of bearings (θ), bi-modal distribution of turning angles ($\Delta\theta$), or composite movement pattern. The panels show: (a) rose diagram of θ, (b) θ over time, (3) $\Delta\theta$ over time, (4) $\Delta\theta$ density distribution, (5) moving window of $\hat{R}_{\theta}$ (window's length =4 steps), (6) moving window of $\hat{R}_{\Delta\theta}$ (window's length =4 steps). Horizontal red lines represent the inter-percentile range (5-95%).

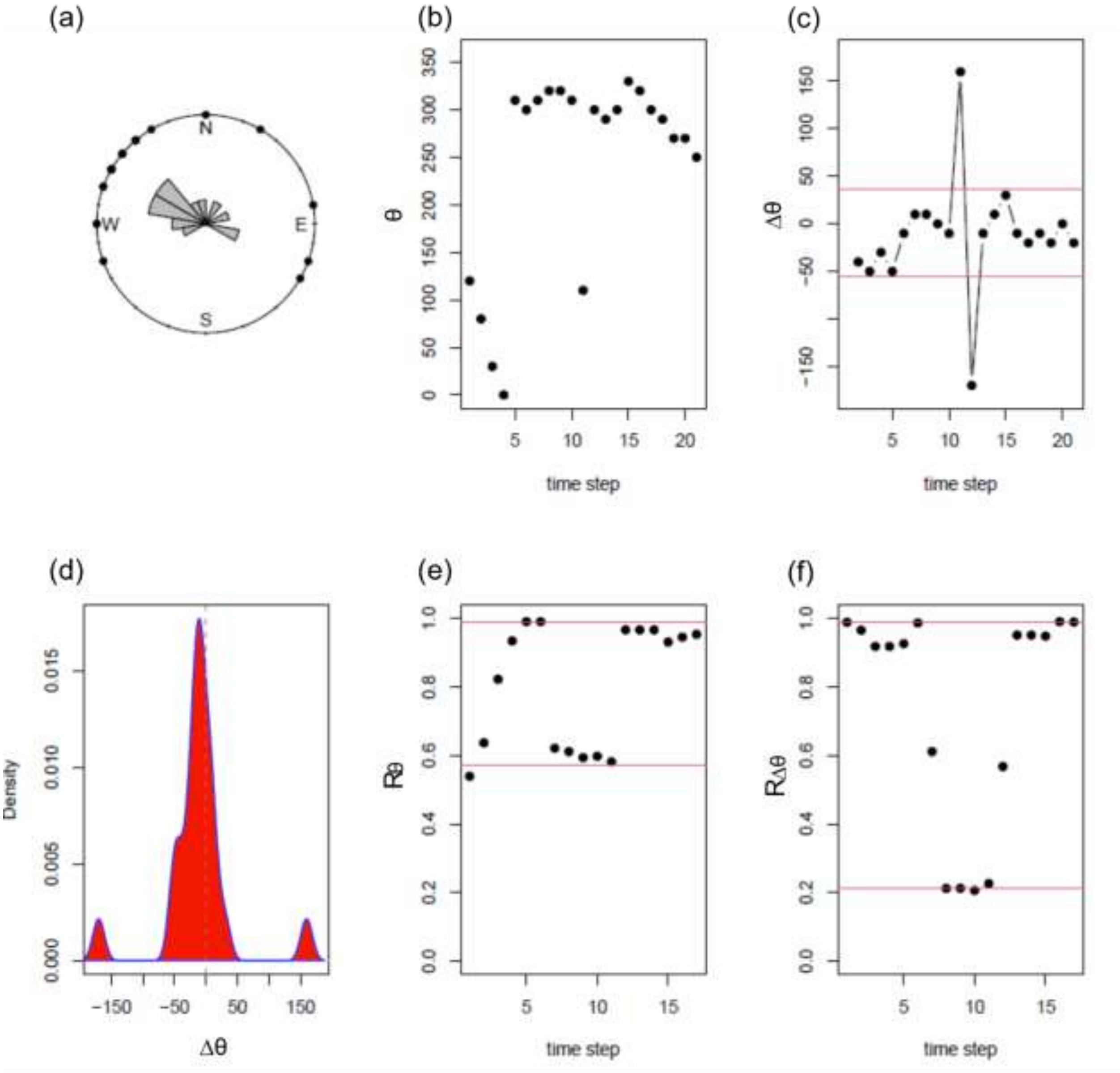

Figure S7. Visual inspection for detecting irregular behaviors- an example that shows irregular patterns. An example of a trial (*Chaetodon aureofasciatus, scuba-following*) that shows several irregular patterns: (1) one-sided bias towards left turns, multi-modal distribution of bearings (θ) (top left panel), and composite movement pattern in which movement precision is not homogenous and changes substantially between different stages of the trial. The panels show: (a) rose diagram of θ, (b) θ over time, (3) turning angles ($\Delta\theta$) over time, (4) $\Delta\theta$ density distribution, (5) moving window of $\hat{R}_{\theta}$ (window's length =4 steps), (6) moving window of $\hat{R}_{\Delta\theta}$ (window's length =4 steps). Horizontal red lines represent the inter-percentile range (5-95%).

Table S1 Simulation details for simulated examples of classic and irregular patterns (Supplementary note 4). Common parameters for all simulations include $kappa\_{CRW}$ =1, 3, 5, 10, 30, number of replicates=1000, number of observations Nobs=21 and 180 for Fig. S4a and S4b, respectively.

Scenario name	Description
CRW	Correlated Random Walk with turning angles drawn from a von Mises distribution centered around zero and $kappa\_{CRW}$ values of 1, 3, 5, 10, 30
BRW	Biased Random Walk with bearings drawn from a von Mises distribution centered around zero and $kappa\_{BRW}$ values of 1, 3, 5, 10, 30
BCRW	Biased Correlated Random Walk with turning angles drawn from a von Mises distribution centered around zero and $kappa\_{CRW}$ values of 1, 3, 5, 10, 30. and re-orientation towards the fixed direction. Re-orientation occurs 0˚every 3 and 22 steps for Nobs=21 and 180, respectively, and includes drawing a heading from a von Mises distribution centered around zero with $kappa\_{BRW}$=3, and adding an error term in form of a turning angle drawn from the CRW von Mises distribution above.
Zig zag	Correlated Random Walk with turning angles drawn from two von Mises distributions centered around positive (30˚) and negative (-30˚) directions and $kappa\_{CRW}$ values of 1, 3, 5, 10, 30.
CRW composite	A sequence that is composed of two types of CRW patterns, alternating half-way through the sequence (i.e., after Nobs/2 steps). The first type includes turning angles drawn from a von Mises distribution with $kappa\_{CRW}$ values of 1, 3, 5, 10, 30 and the second type includes factoring the $kappa\_{CRW}$ by 20, i.e., values of 20, 60, 100, 200, 600. Turning angles distributions of both types are centered around zero.
One-sided $\Delta\theta$ bias	Correlated Random Walk with turning angles drawn from a von Mises distribution centered around 10˚ and $kappa\_{CRW}$ values of 1, 3, 5, 10, 30
