# Supplementary material for: Evidence for a consistent use of external cues by marine fish larvae for orientation
Source: Commun Biol. 2022 Dec 2;5:1307. doi: 10.1038/s42003-022-04137-7 (PMC9718780; doi:10.1038/s42003-022-04137-7)
Supplement: Supplementary file 2 — Description of Additional Supplementary Files [file 42003_2022_4137_MOESM2_ESM.pdf]

Description of Additional Supplementary Files

File name: Supplementary Data S1
Description: Sample dataset of *Caesio cuning*.

File name: Supplementary Code S1
Description: R code with the analyses provided in the paper.
