# Supplementary material for: Evidence for a consistent use of external cues by marine fish larvae for orientation
Source: Commun Biol. 2022 Dec 2;5:1307. doi: 10.1038/s42003-022-04137-7 (PMC9718780; doi:10.1038/s42003-022-04137-7)
Supplement: Supplementary file 5 — Reporting Summary [file 42003_2022_4137_MOESM5_ESM.pdf]

nature research

Corresponding author(s): Claire Paris
Last updated by author(s): Jun 23, 2022

Reporting Summary

Nature Research wishes to improve the reproducibility of the work that we publish. This form provides structure for consistency and transparency in reporting. For further information on Nature Research policies, see our Editorial Policies and the Editorial Policy Checklist.

Statistics

For all statistical analyses, confirm that the following items are present in the figure legend, table legend, main text, or Methods section.

n/a	Confirmed	
☐	☒	The exact sample size (*n*) for each experimental group/condition, given as a discrete number and unit of measurement
☐	☒	A statement on whether measurements were taken from distinct samples or whether the same sample was measured repeatedly
☐	☒	The statistical test(s) used AND whether they are one- or two-sided<br>*Only common tests should be described solely by name; describe more complex techniques in the Methods section.*
☒	☐	A description of all covariates tested
☐	☒	A description of any assumptions or corrections, such as tests of normality and adjustment for multiple comparisons
☐	☒	A full description of the statistical parameters including central tendency (e.g. means) or other basic estimates (e.g. regression coefficient) AND variation (e.g. standard deviation) or associated estimates of uncertainty (e.g. confidence intervals)
☐	☒	For null hypothesis testing, the test statistic (e.g. F, t, r) with confidence intervals, effect sizes, degrees of freedom and P value noted<br>*Give P values as exact values whenever suitable.*
☒	☐	For Bayesian analysis, information on the choice of priors and Markov chain Monte Carlo settings
☒	☐	For hierarchical and complex designs, identification of the appropriate level for tests and full reporting of outcomes
☐	☒	Estimates of effect sizes (e.g. Cohen's *d*, Pearson's *r*), indicating how they were calculated

Our web collection on statistics for biologists contains articles on many of the points above.

Software and code

Policy information about availability of computer code

Data collection	This is a meta-analysis based on data from previously published studies.
Data analysis	The R code for running the analyses was uploaded uploaded to the online system.

For manuscripts utilizing custom algorithms or software that are central to the research but not yet described in published literature, software must be made available to editors and reviewers. We strongly encourage code deposition in a community repository (e.g. GitHub). See the Nature Research guidelines for submitting code & software for further information.

Data

Policy information about availability of data

All manuscripts must include a data availability statement. This statement should provide the following information, where applicable:

- Accession codes, unique identifiers, or web links for publicly available datasets
- A list of figures that have associated raw data
- A description of any restrictions on data availability

The figures associated with the uploaded data are Figures 1 and Figure S1-7. The quantitative protocol is explained in the methods section and appears as comments in the R code file.

Field-specific reporting

Please select the one below that is the best fit for your research. If you are not sure, read the appropriate sections before making your selection.

☐ Life sciences ☐ Behavioural & social sciences ☒ Ecological, evolutionary & environmental sciences

For a reference copy of the document with all sections, see nature.com/documents/nr-reporting-summary-flat.pdf

Ecological, evolutionary & environmental sciences study design

All studies must disclose on these points even when the disclosure is negative.

Study description	We apply a cross-species meta-analysis (of previously published studies), focusing on the fundamental question of whether larval fish use external cues for directional movement. We compare the observed directional patterns to those expected under a strict use of internal cues, and find support for the use of external cues for orientation across species and locations by fish larvae.
Research sample	The raw data include heading sequences of fish larvae from various species, locations, and times.
Sampling strategy	The data, which is based in published studies, is composed of in situ orientation trials, and can be divided into two main categories. First, with direct observations through Scuba-Following, where a larva is released in the pelagic environment and tracked by scuba divers for 10 min during which swimming direction is recorded every 30 s, resulting in 21 observations (Nobs=21). Second, with observations using the Drifting In Situ Chamber (DISC). For each DISC trial, a larva is placed into a circular chamber, and its position is recorded for 15-20 min with a camera fixed circa 50 cm below the chamber. The first 3-5 minutes of each DISC trial are considered as acclimation time and are excluded from the analysis, whereas the residual 10-15 min are the actual observations used for the analysis. It is noteworthy that these two methods have been tested together and have demonstrated high consistency in larval orientation results.
Data collection	Data is based on previously published orientation trials (explained above), and consists of heading sequences of larval fish.
Timing and spatial scale	The studies of which experimental data was used were from various tropical and warm-water locations: East Asia, Australia, Mediterranean and Red Sea (Table 1 in the manuscript). These trials spanned from the late 90s to 2015.
Data exclusions	Data excluded from the analysis if the number of observations per trial was insufficient or if the trial did not have a significant mean vector based on Rayleigh's test (see methods section for more details).
Reproducibility	The analysis can be easily reproduced based on the code provided
Randomization	Randomization was applied in several components of the study: (1) when generating the null distribution in the CRW-vm method, we sample turning angles (with replacement) from a von-Mises distribution (see methods sections). (2) in the CRW re-sampling (CRW-r) analysis, we compute the empirical angle changes (turning angles) per trial, and re-sample them (without replacement) to produce new angle changes sequences, based upon which the null CRW-r is computed (see methods section).
Blinding	n/a

Did the study involve field work? ☐ Yes ☒ No

Reporting for specific materials, systems and methods

We require information from authors about some types of materials, experimental systems and methods used in many studies. Here, indicate whether each material, system or method listed is relevant to your study. If you are not sure if a list item applies to your research, read the appropriate section before selecting a response.

Materials & experimental systems

n/a	Involved in the study
☒	☐ Antibodies
☒	☐ Eukaryotic cell lines
☒	☐ Palaeontology and archaeology
☒	☐ Animals and other organisms
☒	☐ Human research participants
☒	☐ Clinical data
☒	☐ Dual use research of concern

Methods

n/a	Involved in the study
☒	☐ ChIP-seq
☒	☐ Flow cytometry
☒	☐ MRI-based neuroimaging
